# Parent–Child Associations of Eating Habits According to Domains of Parental Physical Activity (EPI-Family Health Study)

**DOI:** 10.3390/nu17203234

**Published:** 2025-10-15

**Authors:** Ewerton P. Antunes, William R. Tebar, Bruna T. C. Saraiva, Amanda Barbosa dos Santos, Stefany C. B. Silva, Débora T. Furuta, Vandrize Meneghini, Jorge Mota, Gerson Ferrari, Diego G. D. Christofaro

**Affiliations:** 1 Postgraduate Program in Movement Sciences, School of Technology and Sciences, São Paulo State University (UNESP), Presidente Prudente 19060-900, SP, Brazil; ewerton.p.antunes@unesp.br (E.P.A.); brunatcsaraiva@gmail.com (B.T.C.S.); amanda.b.santos@unesp.br (A.B.d.S.); stefany.carolaine@unesp.br (S.C.B.S.); debora.t.furuta@unesp.br (D.T.F.); diego.christofaro@unesp.br (D.G.D.C.); 2 Center for Clinical and Epidemiological Research, University Hospital, University of São Paulo (USP), São Paulo 05508-000, SP, Brazil; vandrize@gmail.com; 3 CIAFEL—The Research Centre in Physical Activity, Health and Leisure, University of Porto, 4099-002 Porto, Portugal; jmota@fade.up.pt; 4 Faculty of Health Sciences, Universidad Autónoma de Chile, Providencia 7500912, Chile; gersonferrari08@yahoo.com.br; 5 Sciences of Physical Activity, Sports and Health School, University of Santiago of Chile (USACH), Santiago 7500618, Chile

**Keywords:** eating habits, physical activity, parent–child dyads, family environment, food consumption

## Abstract

**Background**: The family environment and physical activity (PA) levels are known to influence the eating habits of children and adolescents, but it is unclear how different domains of parental PA can affect parent–child associations with eating habits. **Methods**: This study included 473 participants: 192 children and adolescents (aged between 5 and 17 years), 163 mothers, and 118 fathers. Parental PA was assessed in occupational, sports, and leisure-time/commuting domains. Children’s and parents’ dietary intake was evaluated using a weekly food frequency questionnaire, covering healthy and unhealthy food groups. The mother–child and father–child associations were analyzed using multiple quantile regression. **Results**: Active mothers in occupational PA showed mother–child associations for fruits, vegetables, and dairy foods, whereas inactive mothers showed associations for fried foods and sweets. In the sports practice domain, active mothers showed mother–child associations for vegetables and red meat consumption, whereas active fathers showed associations for grains and salty snacks consumption. Active mothers in the leisure-time/commuting domain showed mother–child associations for fried foods and red meat consumption, while active fathers showed associations for fruits and salty snacks consumption. **Conclusions**: Mother–child associations were more consistent across PA domains than father–child associations. Overall, the healthy eating habits of physically active mothers were more strongly linked to the healthy eating patterns of their children.

## 1. Introduction

High consumption of vegetables, fruits, and grains, combined with low intake of ultra-processed foods, such as sweets, snacks, and fast foods, is considered a marker of healthy eating across diverse populations [1,2]. Dietary habits have been associated with various health outcomes in the adult population. For instance, a prospective study with more than 30 years of follow-up observed that healthier eating patterns were associated with an approximately 10% reduction in the risk of cardiovascular disease [3]. Similarly, a meta-analysis of 17 studies, involving 159,885 participants, found that junk food consumption was associated with a higher risk of depression [4].

The relationship between eating habits and health problems has also been observed in younger populations. In a meta-analysis, Cunha et al. [5] reported that adolescents with unhealthy eating habits had a higher body mass index (BMI) and greater abdominal obesity compared to their peers with healthier diets. Likewise, Mesas et al. [6] found that high intake of processed foods was associated with adverse mental health. Conversely, Jonsson et al. [7] showed that increased fruit and vegetable intake was positively associated with improved mental health and well-being in Swedish adolescents.

Two main factors are considered to influence the eating habits of children and adolescents. The first is the family environment. Several studies have shown that parents’ eating habits are associated with those of their children [8,9]. The second factor is physical activity, another important health-related behavior that may be linked to dietary habits in both adult and pediatric populations. Christofaro et al. [10], in a study of 1874 adults during the COVID-19 pandemic, reported that physical activity was associated with healthy eating habits. Similarly, in a study conducted with adolescents in Germany, the authors [11] found that physical activity was associated with higher consumption of water, dairy products, fruits, and vegetables.

Despite these contributions, important gaps remain in the literature. Most previous studies have examined only total physical activity in relation to eating habits [9,12], without accounting for specific physical activity domains, which can be categorized as occupational, sports, and leisure-time/commuting activities. Occupational physical activities are those performed in the workplace or during household chores, such as cleaning the house, washing dishes, making the bed, and others. Sports practice refers to voluntary, structured activities, usually associated with enjoyment, such as playing football, attending a gym, and others. Leisure-time/commuting activities include activities such as walking children to school or going shopping on foot.

Considering these aspects, the different domains of physical activity may be differentially associated with eating habits. Opper et al. [13] in a study of adults in France, found that leisure-time physical activity was associated with increased frequency of fruit consumption, whereas occupational activities were not related to eating habits. One possible explanation for this finding is that individuals who engage in physical activity during leisure time generally do so voluntarily and in a planned manner, and they may also have greater health awareness, which extends to their eating habits. Moreover, in a literature review of 16 articles, Tanaka et al. [14] observed that high levels of occupational activity were associated with poorer diet quality, often characterized by greater consumption of “fast” foods. Findings regarding active commuting are inconsistent. For example, Smith et al. reported that active commuting was positively associated with healthier eating habits in a multicenter study across the UK, while Silva et al. [15] in a study of more than 50,000 Brazilians, found no such association. Cultural differences, as well as the time and quality of commuting, may partly explain these discrepancies.

Within this context, the present study aims to address an important gap. To our knowledge, this is the first investigation to examine parent–child similarities in eating habits stratified by different domains of parental physical activity. We hypothesized that the strength and direction of parent–child eating habit associations vary across physical activity domains.

Therefore, the objective of the current study was to analyze the associations between parents’ and children’s eating habits, stratified by parental PA domains, while adjusting for covariates including sex, age, socioeconomic status, and the PA level of the children and adolescents.

## 2. Materials and Methods

### 2.1. Sample

This cross-sectional study was approved by the Research Ethics Committee of FCT-UNESP (FCT-UNESP) under protocol CAAE 59261422.2.0000.5402. Children and adolescents aged 6–17 years, along with at least one parent, were invited to participate. This project, entitled the EPI-FAMILY HEALTH STUDY [16], aimed to investigate the associations between parents’ and children’s lifestyle habits, focusing particularly on the influence of parents’ physical activity and eating habits. Families from different regions of the city of Presidente Prudente, São Paulo (north, south, east, west, and central areas) were included. Eligible children and adolescents were enrolled in public or private schools in the city. Recruitment was carried out through social media and flyers distributed at events/fairs, schools, and gyms in the city. The sample included 473 participants: 192 children and adolescents, 163 mothers, and 118 fathers; these families covered all regions of the city of Presidente Prudente in 2023/2024. Data collection took place on the FCT-UNESP premises, where all participants signed an assent/consent form and received a briefing about the study.

Inclusion criteria were: (i) ability to understand the questionnaires administered, and (ii) ability to use the accelerometer. The exclusion criteria were: (i) failure to use the accelerometer for the minimum amount of time; and (ii) incomplete or inconsistent questionnaire responses.

The sample size was estimated using a correlation coefficient of 0.29 for fruit and vegetable intake, based on the study by Beydon et al. [17], which investigated the parent–child relationship. An alpha error of 5% was adopted, with a sample power of 80%. To prevent sample losses, an additional 10% was added to the calculation, resulting in 101 children and at least one parent (yielding a total of 202 participants).

### 2.2. Eating Habits

Eating habits were assessed using a weekly food frequency questionnaire, adapted from Block et al. [18]. This questionnaire included food groups related to: healthy habits, such as fruits, vegetables, dairy products, grains, and red meat; and unhealthy eating habits, such as fried foods, sweets, fast food, soft drinks, and salty snacks. The frequency of food consumption was analyzed as days per week (0–7). The questionnaire by Block et al. has been validated for the adult [18] and adapted/validated for the pediatric population [19]. We tested the reliability of the questionnaire in a subsample; participants completed the questionnaire twice with a two-week interval. Intraclass correlation coefficient values ranging from 0.73 to 1.00 were found.

### 2.3. Parental Physical Activity

Parental physical activity domains were assessed using the Baecke Habitual Physical Activity Questionnaire [20]. This instrument consists of 16 items and provides dimensionless scores based on a 12-month recall. The questionnaire is divided into three domains: occupational, sports practice, and leisure-time/active commuting, and assess: (i) occupational physical activity, with questions investigating the physical effort required in the workplace, considering postures, commuting, and activities performed during the workday; (ii) sports practice, taking into account weekly frequency, average duration, and intensity of the activity; (iii) physical activity during commuting and leisure-time, addressing habits related to commuting by walking and cycling, as well as sedentary behavior in leisure through watching television.

Each item was scored based on a Likert-type scale (ranging from 1 to 5), with scores used to calculate an index for each domain. These scores can also be added together to obtain an overall physical activity index, with an approximate range of 3 to 15 points.

The questionnaire provides a score for each of the three domains of physical activity practiced by participants. Given the instrument’s dimensionless nature, participants were classified into tertiles to compare the highest tertile (defined as physically active) against the lower tertiles (physically inactive) in each domain.

The questionnaire was chosen due to its widespread use in epidemiological studies, as well as its validation for Brazilian adults [21]. This instrument presents good reliability rates for both adults (ICC: 0.68–0.97) and children/adolescents (ICC: 0.65–0.85).

### 2.4. Covariates

The sex, age, physical activity, and socioeconomic status of the children/adolescents were considered covariates in the present study. Physical activity was assessed using accelerometry, aiming to quantify which children did or did not reach the recommended amount of moderate/vigorous physical activity (60 min/day). To this end, the children and adolescents were instructed to wear the Actigraph wGT3X-BT accelerometer for seven full days, removing the device for water activities/hygiene and nighttime sleep. The accelerometers were programmed with a capture rate of 30 Hz and an epoch of 60 s. Actilife 6.13.1 software was utilized for data analysis, using the cut-off point proposed by Evenson et al. [22]. The minimum wear time for validation was four days, one of which was on the weekend, with at least 10 h of daily use each day.

To classify the socioeconomic status of the participants, we used the questionnaire from the Brazilian Association of Research Companies—ABEP [23]. The instrument was administered via a face-to-face interview to participants, and they were classified into one of six socioeconomic classes: A, B1, B2, C1, C2, or DE. The questionnaire covers information such as the education level of the head of the household (i.e., the person who contributes the majority of the family income), the number of assets and rooms in the residence, and the number of vehicles available. The presence and number of certain assets and rooms in the home, the source of the water consumed (whether the household has running water or not), and the condition of the road where the residence is located (whether paved or not) were also investigated. To analyze the reliability of this instrument, a portion of the sample answered the questionnaire twice with a two-week interval, and the ICC values observed were considered good, ranging from 0.72 to 1.00.

### 2.5. Statistical Analysis

The descriptive characteristics for the children, mothers, and fathers are expressed as medians and interquartile ranges due to non-normal distributions. Quantile Regression (adopted considering the “non-normal” distribution of data) was used to assess the relationships between parental and child eating habits, considering parental eating habits as predictors and children’s habits as outcomes. Results are expressed as regression coefficients representing changes in the median (50th percentile) outcome per unit increase in the median predictor. Analyses were stratified by parent sex (mother–child vs. father–child dyads) and by parental physical activity status (active vs. inactive). Adjustment variables included the sex, age, and physical activity of the children, as well as parents’ age, socioeconomic status, and education. The significance level was set at *p* < 0.05, with 95% confidence intervals. Statistical analyses were performed using SPSS, version 29.0.

## 3. Results

The current study included 473 participants: 163 mothers, 118 fathers, and 192 children (39.6% girls). Overall, all participants reported a high frequency of grain intake, with a median of seven days a week. Among unhealthy foods, salty snacks showed the lowest consumption, followed by soft drinks, fast food, and fried foods. Children and adolescents had a median value of 192.5 (P25: 115.5; P75: 303.0) minutes of moderate-to-vigorous physical activity per week. The sample characteristics are presented in Table 1.

Parental physical activity domain scores were compared according to whether parents were classified as physically active or inactive within a specific domain. Mothers active in the occupational domain showed higher scores in sports (3.2 [1.8–4.4] vs. 2.5 [1.0–4.1]; *p* = 0.018) and leisure-time/commuting (2.3 [2.0–2.8] vs. 1.8 [1.5–2.3]; *p* < 0.001) than inactive mothers. Mothers active in the sports domain did not differ in occupational activity (2.6 [1.9–2.9] vs. 2.3 [1.5–3.0]; *p* = 0.072), but had higher levels of physical activity in leisure-time/commuting (2.5 [2.0–3.0] vs. 1.8 [1.5–2.3]; *p* < 0.001). Mothers active in leisure-time/commuting showed higher scores in the occupational (2.6 [1.9–3.4] vs. 1.9 [1.0–2.8]; *p* < 0.001) and sports domains (3.6 [2.0–5.2] vs. 1.8 [1.0–3.3]; *p* < 0.001) than inactive mothers. For fathers, those active in the occupational domain did not differ from inactive fathers in sports (3.3 [2.0–5.0] vs. 4.2 [2.0–5.1]; *p* = 0.414) or leisure-time/commuting activity (2.3 [2.0–2.5] vs. 2.3 [2.0–2.8]; *p* = 0.733). Fathers active in the sports domain showed similar occupational activity (2.2 [1.9–2.8] vs. 2.3 [1.6–2.8]; *p* = 0.894), but higher levels in leisure-time/commuting (2.5 [2.3–3.0] vs. 2.0 [1.8–2.5]; *p* < 0.001). Fathers active in leisure-time/commuting did not differ in occupational activity (2.3 [1.9–3.0] vs. 2.3 [1.6–2.8]; *p* = 0.295), but had higher scores in sports (4.5 [3.4–5.6] vs. 2.9 [1.3–4.6]; *p* < 0.001), when compared to inactive fathers.

Table 2 presents the parent–child associations with eating habits according to parental physical activity in the occupational domain, stratified by mothers and fathers. Significant mother–child associations were observed among inactive mothers for fried foods (β = 0.470; *p* = 0.017), sweets (β = 0.554; *p* = 0.005), fast foods (β = 0.305; *p* = 0.032), and red meat (β = 0.496; *p* = 0.961). Among active mothers, significant associations were found for fruits (β = 0.398; *p* = 0.013), vegetables (β = 0.833; *p* = 0.013), dairy products (β = 0.600; *p* < 0.001), fried foods (β = 0.442; *p* = 0.013), soft drinks (β = 0.313; *p* = 0.039), salty snacks (β = 0.467; *p* = 0.039), and red meat (β = 0.645; *p* = 0.011). In contrast, inactive fathers in the occupational domain showed significant father–child associations for fruits (β = 0.524; *p* = 0.011), vegetables (β = 0.670; *p* = 0.010), sweets (β = 0.504; *p* = 0.043), and soft drinks (β = 0.549; *p* = 0.001), while no significant associations were observed for active fathers.

Regarding parental physical activity in the sports domain (Table 3), inactive mothers showed significant mother–child associations for fruits (β = 0.273; *p* = 0.026), vegetables (β = 0.892; *p* < 0.001), and fried foods (β = 0.474; *p* = 0.004). Active mothers showed significant associations for vegetables (β = 0.853; *p* = 0.011), salty snacks (β = 1.495; *p* < 0.001), and red meat (β = 0.870; *p* < 0.001). Inactive fathers in the sports domain showed significant father–child associations for fruits (β = 0.675; *p* = 0.010), soft drinks (β = 0.331; *p* = 0.001), and red meat (β = 0.623; *p* = 0.011), whereas active fathers showed significant associations for grains (β = 0.714; *p* < 0.001), fast foods (β = 0.418; *p* = 0.035), and salty snacks (β = 0.812; *p* < 0.001).

Table 4 presents the associations between parental and child eating habits according to parental physical activity in the leisure-time/commuting domain. Physically inactive mothers showed significant associations for fast foods (β = 0.375; *p* = 0.006) and soft drinks (β = 0.511; *p* < 0.001). Active mothers showed associations for fruits (β = 0.420; *p* = 0.029), fried foods (β = 0.442; *p* = 0.002), fast foods (β = 0.269; *p* = 0.009), and red meat (β = 0.743; *p* = 0.001). Inactive fathers showed significant father–child associations for fried foods (β = −0.390; *p* = 0.009), fast food (β = 0.275; *p* = 0.013), and soft drinks (β = 0.289; *p* = 0.040). Among active fathers, significant associations were observed for fruits (β = 0.807; *p* < 0.001), soft drinks (β = 0.414; *p* = 0.003), and salty snacks (β = 0.636; *p* < 0.001).

## 4. Discussion

The current study investigated the relationship between the eating habits of parents and children across different domains of parental physical activity, namely occupational, sports, and leisure-time/commuting activities. We found consistent positive associations between the eating habits of parents and their children; however, the strength and pattern of these associations varied according to the parental sex and physical activity level. Stratified analyses revealed that active and inactive mothers and fathers showed different dietary similarities with their children. Overall, mothers exhibited broader associations across both healthy and unhealthy food groups, whereas fathers tended to show more selective patterns. These findings suggest that parental physical activity interacts with sex in shaping the food groups where parent–child similarities are most evident.

In the occupational domain, stronger associations with healthy food consumption were observed in mother–child dyads when mothers were physically active. For unhealthy foods, the associations between mothers and children were similar regardless of maternal physical activity status. In contrast, in the father–child relationship, the strongest associations for both healthy and unhealthy foods were observed among inactive fathers. One possible explanation for these findings is that physically active mothers are more likely to adopt other health-promoting behaviors, such as higher fruit, vegetable, and grain consumption. According to the meta-analysis by Yee et al. [24], factors such as greater educational guidance and moderate maternal influence could contribute to more healthy habits in children. It should also be noted that mothers, particularly those working at home, are often responsible for preparing family meals. For fathers, the type of physical effort in occupational physical activity (for example, heavier physical activities while exposed to the sun) may lead to greater fatigue and sedentary behavior during leisure time. This sedentary behavior may be shared with children, increasing screen time and, consequently, exposure to unhealthy foods such as snacks and fast foods [25,26].

In the sports domain, associations between parent–child unhealthy food consumption were observed in both inactive mothers and fathers. This aligns with evidence that individuals who engage in physical activity tend to have healthier eating habits [27]. Monserrat-Mesquida et al. [28] observed that higher parental physical activity was associated with lower consumption of fast food and candy among children. A possible mechanism underlying these findings is that physical activity may mitigate anxiety, thereby reducing the consumption of ultra-processed foods. A study conducted during the COVID-19 pandemic showed that anxiety was more closely related to poor eating habits in physically inactive participants compared with active participants [29]. This relationship can be extrapolated to the family environment, as Ghazy Elsayed et al. [30] observed that adolescents who felt they were in an emotionally stable and welcoming family environment were more likely to consume fruits and vegetables.

In the leisure-time/active commuting domain, the mother–child associations for healthy food were stronger among active mothers, while those for unhealthy food were similar between inactive and active mothers. Similar findings were observed for healthy food consumption among active fathers and their children. However, for unhealthy food, the associations were slightly stronger among inactive fathers. Several mechanisms may explain these findings. First, people who are more active in leisure time and commuting may be less exposed to fast food, which is often found in convenience stores at gas stations [31]. Second, physical activities such as walking or cycling may contribute to the regulation of appetite-related hormones like ghrelin and leptin [32]. Supporting this, Smith et al. [33], in a large study using data from the UK Biobank, found that active lifestyle behaviors were associated with healthier dietary choices. It is possible that parents engaged in active commuting may also maintain more structured daily routines, which can include preparing healthier meals for both work and school.

As limitations of this study, the cross-sectional design prevents the analysis of cause-and-effect relationships. We used a questionnaire that assesses only the weekly frequency of food consumption, not a dietary recall, so it was not possible to quantify through portion size or total nutrient intake. The amount of time that parents and children spent together was not assessed, which may influence shared eating behaviors. Additionally, the initial sample size calculation was not assured for this specific research question and cannot have been specifically powered for all the hypothesis due to be a secondary analysis and a post hoc power analyses are not appropriate for quantile regression models, since power estimation in this context depends on the conditional distribution of the response variable. Despite these limitations, we highlight that the analyses considered both mothers’ and fathers’ behavior and the final sample (*n* = 473) was 2.3-fold larger than the minimum required, with the analysis across distinct domains of physical activity being another innovative aspect. To our knowledge, this is the first study to stratify father–child dietary associations according to different domains of paternal physical activity.

## 5. Conclusions

The strongest parent–child associations in eating habits were observed in the occupational domain, particularly when mothers were physically active. Associations were also present, although to a lesser extent, in the leisure-time/commuting domain. In commuting activities, mother–child associations were more evident among active mothers, while father–child associations were comparable regardless of paternal activity status. Overall, parental physical activity positively influenced parent–child eating habits, especially in the mother–child dyad. These findings confirm our hypothesis that the relationships between parent–child eating habits tend to vary according to the physical activity domain investigated.

## Figures and Tables

**Table 1:** Characteristics of the sample.

	Children Median (P25–P75)	Mothers’ Median (P25–P75)	Fathers’ Median (P25–P75)
Age (years)	10.0 (8.0–14.0)	42.0 (37.0–45.0)	44.0 (40.0–47.0)
BMI (kg/cm^2^)	18.8 (16.4–21.4)	25.7 (23.4–29.6)	28.5 (25.8–32.2)
MVPA (minutes/week)	192.5 (115.5–303.0)	71.5 (36.3–140.0)	137.0 (66.3–222.0)
Eating habits			
Fruits (days/week)	4.0 (2.0–7.0)	5.0 (3.0–7.0)	3.0 (2.0–5.0)
Vegetables (days/week)	4.0 (1.0–7.0)	5.0 (4.0–7.0)	5.0 (3.0–6.0)
Dairy (days/week)	5.0 (3.0–7.0)	4.0 (2.0–7.0)	3.0 (1.0–6.0)
Grains (days/week)	7.0 (7.0–7.0)	6.0 (5.0–7.0)	7.0 (5.0–7.0)
Fried foods (days/week)	2.0 (1.0–3.0)	1.0 (0.0–2.0)	1.0 (1.0–2.0)
Sweets (days/week)	4.0 (2.0–7.0)	3.0 (2.0–5.0)	2.0 (1.0–4.0)
Fast foods (days/week)	1.0 (1.0–2.0)	1.0 (1.0–2.0)	1.0 (1.0–2.0)
Soft drinks (days/week)	2.0 (0.0–3.0)	1.0 (0.0–2.0)	1.0 (0.0–3.0)
Salty snacks (days/week)	1.0 (0.0–2.0)	0.0 (0.0–1.0)	0.0 (0.0–0.0)
Red meat (days/week)	4.0 (2.0–6.0)	4.0 (3.0–5.0)	4.0 (3.0–5.0)

BMI = Body Mass Index; MVPA = Moderate-to-vigorous physical activity.

**Table 2:** Associations of eating habits according to parental physical activity in the occupational domain.

	Physically Inactive Mothers				Physically Active Mothers			
	β	95% CI	*p*	R^2^q	β	95% CI	*p*	R^2^q
Fruits	0.318	−0.022; 0.659	0.066	0.095	0.398	0.088; 0.709	0.013	0.188
Vegetables	0.385	−0.158; 0.928	0.161	0.071	0.833	0.273; 1.393	0.004	0.139
Dairy	−0.103	−0.384; 0.179	0.468	0.084	0.600	0.274; 0.926	<0.001	0.152
Grains	0.000	−0.001; 0.001	0.622	0.000	0.000	−0.001; 0.001	0.279	0.000
Fried foods	0.470	0.088; 0.852	0.017	0.099	0.442	0.098; 0.786	0.013	0.089
Sweets	0.554	0.169; 0.939	0.005	0.141	−0.007	−0.479; 0464	0.975	0.155
Fast foods	0.305	0.027; 0.583	0.032	0.135	0.000	−0.186; 0.186	0.999	0.000
Soft drinks	−0.106	−0.356; 0.144	0.400	0.090	0.313	0.017; 0.609	0.039	0.053
Salty snacks	0.508	−0.137;1.154	0.120	0.057	0.467	0.024; 0.909	0.039	0.108
Red meat	0.496	0.030; 0.961	0.037	0.144	0.645	0.157; 1.133	0.011	0.165
	**Physically Inactive Fathers**				**Physically Active Fathers**			
Fruits	0.524	0.127; 0.921	0.011	0.166	0.045	−0.521; 0.612	0.870	0.211
Vegetables	0.670	0.165; 1.175	0.010	0.140	0.188	−0.429; 0.806	0.535	0.091
Dairy	0.065	−0.317; 0.448	0.734	0.088	−0.162	−0.540; 0.215	0.385	0.110
Grains	0.000	−0.001; 0.001	0.841	0.000	0.000	−0.209; 0.209	0.999	0.000
Fried foods	0.051	−0.285; 0.387	0.764	0.038	−0.398	−1.095; 0.298	0.250	0.164
Sweets	0.504	0.018; 0.990	0.043	0.104	−0.153	−0.828; 0.522	0.644	0.075
Fast foods	0.291	−0.014; 0.596	0.061	0.057	0.147	−0.236; 0.530	0.436	0.053
Soft drinks	0.549	0.226; 0.872	0.001	0.175	0.116	−0.125; 0.357	0.329	0.148
Salty snacks	0.257	−0.051; 0.564	0.101	0.063	0.232	−0.600; 1.063	0.571	0.037
Red meat	0.560	−0.009; 1.129	0.054	0.171	−0.074	−0.458; 0.311	0.696	0.186

CI = Confidence Interval; R^2^q = Pseudo R squared for quantile regression. Adjusted by children’s sex, age, socioeconomic status, body mass index, and moderate-to-vigorous physical activity.

**Table 3:** Associations of eating habits according to parental physical activity in the sports practice domain.

	Physically Inactive Mothers				Physically Active Mothers			
	Β	95% CI	*p*	R^2^q	β	95% CI	*p*	R^2^q
Fruits	0.273	0.034; 0.511	0.026	0.092	0.336	−0.225; 0.897	0.234	0.152
Vegetables	0.892	0.436; 1.348	<0.001	0.146	0.853	0.204; 1.501	0.011	0.111
Dairy	0.000	−0.246; 0.246	0.999	0.085	0.022	−0.331; 0.375	0.900	0.128
Grains	0.000	−0.001; 0.001	0.150	0.000	0.000	−0.001; 0.001	0.638	0.000
Fried foods	0.474	0.159; 0.789	0.004	0.102	0.418	−0.246; 1.082	0.211	0.079
Sweets	0.068	−0.384; 0.520	0.765	0.146	0.646	−0.021; 1.313	0.057	0.098
Fast foods	0.132	−0.026; 0.289	0.101	0.060	0.000	−0.592; 0.592	0.999	0.073
Soft drinks	0.204	−0.014; 0.422	0.066	0.039	0.132	−0.340; 0.604	0.577	0.162
Salty snacks	0.253	−0.072; 0.579	0.125	0.053	1.495	0.698; 2.291	<0.001	0.231
Red meat	0.358	−0.145; 0.860	0.160	0.089	0.870	0.423; 1.317	<0.001	0.266
	**Physically Inactive Fathers**				**Physically Active Fathers**			
Fruits	0.675	0.165; 1.184	0.010	0.059	0.314	−0.228; 0.856	0.247	0.143
Vegetables	0.150	−0.492; 0.791	0.642	0.073	0.437	−0.208; 1.082	0.177	0.183
Dairy	0.055	−0.248; 0.358	0.718	0.067	−0.042	−0.554; 0.471	0.870	0.172
Grains	0.000	−0.001; 0.001	0.344	0.000	0.714	0.493; 0.936	<0.001	0.019
Fried foods	−0.139	−0.656; 0.378	0.593	0.029	−0.185	−0.598; 0.228	0.368	0.052
Sweets	0.224	−0.359; 0.807	0.444	0.068	0.252	−0.271; 0.775	0.333	0.141
Fast foods	0.000	−0.202; 0.202	0.999	0.000	0.418	0.030; 0.806	0.035	0.168
Soft drinks	0.331	0.135; 0.528	0.001	0.120	0.302	−0.186; 0.791	0.217	0.152
Salty snacks	−0.052	−0.511; 0.407	0.820	0.003	0.812	0.461; 1.163	<0.001	0.219
Red meat	0.623	0.148; 1.097	0.011	0.190	−0.188	−0.742; 0.367	0.496	0.227

CI = Confidence Interval; R^2^q = Pseudo R-squared for quantile regression. Adjusted by sex, age, socioeconomic status, and children’s moderate/vigorous physical activity.

**Table 4:** Associations of eating habits according to parental physical activity in the leisure-time and commuting domain.

	Physically Inactive Mothers				Physically Active Mothers			
	Β	95% CI	*p*	R^2^q	β	95% CI	*p*	R^2^q
Fruits	0.410	−0.003; 0.822	0.051	0.129	0.420	0.045; 0.795	0.029	0.150
Vegetables	0.403	−0.173; 0.979	0.167	0.146	0.324	−0.148; 0.796	0.175	0.122
Dairy	0.052	−0.305; 0.409	0.772	0.021	0.124	−0.153; 0.402	0.373	0.186
Grains	0.000	−0.001; 0.001	0.878	0.000	0.000	−0.001; 0.001	0.486	0.000
Fried foods	−0.165	−0.720; 0.390	0.554	0.041	0.442	0.163; 0.722	0.002	0.147
Sweets	0.438	−0.053; 0.929	0.079	0.122	0.150	−0.255; 0.555	0.462	0.105
Fast foods	0.375	0.113; 0.638	0.006	0.096	0.269	0.071; 0.467	0.009	0.068
Soft drinks	0.511	0.247; 0.775	<0.001	0.093	−0.015	−0.177; 0.146	0.850	0.111
Salty snacks	0.301	−0.398; 1.001	0.392	0.184	0.397	−0.017; 0.811	0.060	0.047
Red meat	0.642	−1.128; 2.962	0.373	0.121	0.743	0.304; 1.182	0.001	0.209
	**Physically Inactive Fathers**				**Physically Active Fathers**			
Fruits	0.457	0.040; 0.874	0.033	0.119	0.807	0.377; 1.238	<0.001	0.164
Vegetables	0.458	−0.126; 1.042	0.122	0.099	0.500	−0.122; 1.122	0.112	0.174
Dairy	0.000	−0.321; 0.321	0.999	0.059	0.297	−0.043; 0.636	0.085	0.126
Grains	0.000	−0.173; 0.173	0.999	0.000	0.000	−0.001; 0.001	0.067	0.000
Fried foods	−0.390	−0.679; −0.101	0.009	0.065	0.188	−0.392; 0.769	0.516	0.090
Sweets	0.427	−0.137; 0.991	0.134	0.133	0.463	−0.133; 1.058	0.125	0.123
Fast foods	0.275	0.060; 0.491	0.013	0.105	0.000	−0.570; 0.570	0.999	0.028
Soft drinks	0.289	0.014; 0.565	0.040	0.116	0.414	0.149; 0.679	0.003	0.223
Salty snacks	−0.101	−0.490; 0.287	0.602	0.096	0.636	0.286; 0.986	<0.001	0.162
Red meat	0.448	−0.024; 0.919	0.062	0.101	0.120	−0.666; 0.907	0.759	0.170

CI = Confidence Interval; R^2^q = Pseudo R-squared for quantile regression. Adjusted by sex, age, socioeconomic status, and children’s moderate/vigorous physical activity.

## Data Availability

The datasets from the research will be made available by the corresponding author upon an ethically approved proposal and reasonable request.

## Abbreviations

The following abbreviations are used in this manuscript:

PA	Physical activity
BMI	Body mass index
MVPA	Moderate-to-vigorous physical activity
CI	Confidence interval
ABEP	Brazilian Association of Research Companies
